# Microbial and chemical contamination of water, sediment and soil in the Nakivubo wetland area in Kampala, Uganda

**DOI:** 10.1007/s10661-015-4689-x

**Published:** 2015-06-30

**Authors:** Samuel Fuhrimann, Michelle Stalder, Mirko S. Winkler, Charles B. Niwagaba, Mohammed Babu, Godfrey Masaba, Narcis B. Kabatereine, Abdullah A. Halage, Pierre H. H. Schneeberger, Jürg Utzinger, Guéladio Cissé

**Affiliations:** Department of Epidemiology and Public Health, Swiss Tropical and Public Health Institute, P.O. Box, CH-4002 Basel, Switzerland; University of Basel, Basel, Switzerland; Institute for Biogeochemistry and Pollution Dynamics, ETH Zurich, Zurich, Switzerland; Department of Civil and Environmental Engineering, Makerere University, Kampala, Uganda; Department of Research and Development, National Water and Sewerage Corporation, Kampala, Uganda; Vector Control Division, Ministry of Health, Kampala, Uganda; Makerere University School of Public Health, Kampala, Uganda; Department of Epidemiology and Molecular Diagnostics, Agroscope Changins-Wädenswil ACW, Wädenswil, Switzerland; Department of Virology, Spiez Laboratory, Federal Office for Civil Protection, Spiez, Switzerland

**Keywords:** Bacteria, Heavy metals, Helminths, Uganda, Wastewater reuse, Wetland

## Abstract

The reuse of domestic and industrial wastewater in urban settings of the developing world may harm the health of people through direct contact or via contaminated urban agricultural products and drinking water. We assessed chemical and microbial pollutants in 23 sentinel sites along the wastewater and faecal sludge management and reuse chain of Kampala, Uganda. Water samples were examined for bacteria (thermotolerant coliforms (TTCs), *Escherichia coli* and *Salmonella* spp.) and helminth eggs. Physico-chemical parameters were determined. Water, sediment and soil samples and edible plants (yams and sugar cane) were tested for heavy metals. Water samples derived from the Nakivubo wetland showed mean concentrations of TTCs of 2.9 × 10^5^ colony-forming units (CFU)/100 mL. Mean *E. coli* was 9.9 × 10^4^ CFU/100 mL. Hookworm eggs were found in 13.5 % of the water samples. Mean concentrations of iron (Fe), copper (Cu) and cadmium (Cd) were 21.5, 3.3 and 0.14 mg/L, respectively. In soil samples, we found a mean lead (Pb) concentration of 132.7 mg/L. In yams, concentrations of Cd, chromium (Cr) and Pb were 4.4, 4.0 and 0.2 mg/L, while the respective concentrations in sugar cane were 8.4, 4.3 and 0.2 mg/L. TTCs and *E. coli* in the water, Pb in soil, and Cd, Cr and Pb in the plants were above national thresholds. We conclude that there is considerable environmental pollution in the Nakivubo wetland and the Lake Victoria ecosystem in Kampala. Our findings have important public health implications, and we suggest that a system of sentinel surveillance is being implemented that, in turn, can guide adequate responses.

## Introduction

For centuries, humans have reused wastewater to enhance agricultural production (Drechsel et al. 2010). In view of population growth, increasing scarcity of fresh water and the demand to boost food production, reuse of wastewater in agriculture and aquaculture has gained traction in the 21st century (WHO 2006). Particularly in urban and peri-urban areas of low- and middle-income countries, wastewater reuse can support livelihoods of poor communities (Scott et al. 2004). However, contact with untreated wastewater is associated with microbial and chemical hazards and thus can negatively impact human health (Cissé et al. 2002; Matthys et al. 2006). Indeed, pathogenic bacterial and viral organisms can cause diarrhoea, respiratory tract infections, skin and eye diseases and epidemic disease outbreaks such as cholera and typhoid fever (Blumenthal and Peasey 2002; Ensink 2006; Drechsel et al. 2010; Stenström et al. 2011; Becker et al. 2013). Environmental contamination with helminth eggs and intestinal protozoa cyst can drive transmission of intestinal parasitic infections (Matthys et al. 2006, 2007; Ziegelbauer et al. 2012; Pham-Duc et al. 2013; Strunz et al. 2014). Additionally, chronic diseases and cancer are associated with the ingestion and bioaccumulation of heavy metals such as cadmium (Cd) and lead (Pb) or toxic chemicals (e.g. pesticides) discharged in industrial effluents (Jarup 2003; Marcussen et al. 2008; Ackah et al. 2013).

Standardised methods are available to assess and mitigate health risks in connection with the reuse of wastewater, excreta and greywater in agriculture and aquaculture (WHO 2006). However, the practicability and uptake of these methods in low- and middle-income countries proved difficult. Indeed, there is a paucity of quality data that are obtained in a timely manner to guide adequate responses. There is a need for case studies that will deepen our understanding of the level of contamination in wastewater systems, including specific health risks in different exposure groups (Ensink and van der Hoek 2009; Mara et al. 2010; Keraita and Dávila 2015).

In Kampala, Uganda, reuse of wastewater in urban agriculture is commonly practiced, generating important livelihood opportunities for local dwellers in wetland areas (Cole et al. 2008). Approximately 31 km^2^ of the city is classified as wetlands that have an important economic and environmental value for wastewater purification and nutrient retention (Emerton 1998). The largest wetland in Kampala is the Nakivubo system. This wetland is fed from the Nakivubo channel, an open waste and storm water channel, transporting most of the domestic and industrial wastewater of the central division of Kampala (Matagi 2002). The channel also receives secondary treatment effluent from the Bugolobi Sewage Treatment Works (BSTW) and is fed with untreated sewage from informal settlements along the wetland. During the rainy season, the channel and parts of the wetland are often flooded, exposing local residents to raw wastewater (Kayima et al. 2008).

Previous studies reported high concentrations of microbes (thermotolerant coliforms (TTCs)) and toxic chemicals (heavy metals) in the Nakivubo channel, thus posing a risk for deterioration of the surrounding natural ecosystems; namely, the Nakivubo wetland and Lake Victoria (Emerton 1998; Kansiime and Nalubega 1999; Huising 2002; Kayima et al. 2008; Mbabazi et al. 2010). It has been speculated that the natural treatment capacity of the wetland is insufficient for the amount of wastewater, which might be explained by the channelisation of the flow and the reduction of the natural wetland flora as a result of farming activities (Mbabazi et al. 2010). Workers at the wastewater treatment plant, farmers and local communities are at risk of adverse health effects linked to exposure to wastewater and faecal sludge (Nabulo et al. 2006; Katukiza et al. 2014). Additionally, the discharge of contaminated wastewater into the Inner Murchison Bay of Lake Victoria threatens the fishery industry (Birungi et al. 2007) and the drinking water supply of Kampala (Beller et al. 2004; Howard et al. 2006). The intake point of the drinking water supply system for Kampala is located in Ggaba, which is only 4 km from where the Nakivubo channel discharges into Lake Victoria. Moreover, the lake itself is under threat from eutrophication (Focardi et al. 2006).

The Nakivubo area is under considerable pressure due to profound demographic and ecological transformations, including rapid urbanisation, industrial developments and the establishment of informal settlement alongside the Nakivubo wetland (Kayima et al. 2008; Mbabazi et al. 2010). These contextual factors have led to increased volumes of wastewater, putting additional strains on an insufficiently equipped sanitary infrastructure (Beller et al. 2004; Fuhrimann et al. 2014). Hence, there is a need for a sound assessment of relevant environmental, chemical and microbiological parameters along the entire chain from the wastewater treatment plant to Lake Victoria, to enhance evidence-based decision-making for protecting ecosystems and the services that they provide and people’s health and well-being (WHO 2006).

Within the frame of a larger project (Fuhrimann et al. 2014), the objective of the study presented here was to assess faecal and industrial contamination along the major wastewater system in Kampala to identify potential health risks for specific population groups that show different exposures. Thus, physico-chemical parameters, bacterial and helminth contamination and levels of heavy metals were determined in water, sediment, soil and plant samples at 23 sentinel sites.

## Materials and methods

### Ethics statement

The study protocol was approved by the institutional research commission of the Swiss Tropical and Public Health Institute (Swiss TPH; Basel, Switzerland; reference no. FK 106) and the Uganda National Council for Science and Technology (UNCST; Kampala, Uganda; reference no. HS 1487). Ethical approval was obtained from the ethics committee in Basel (EKBB; Basel, Switzerland;reference no. 137/13) and Higher Degrees Research and Ethics Committee of Makerere University School of Public Health (Kampala, Uganda; reference no. IRBOOO11353). This study is registered with the clinical trial registry ISRCTN (identifier: ISRCTN13601686).

### Study area

Kampala is the capital of Uganda with a resident population of about 1.8 million people. The city is located at the northern shores of Lake Victoria at an altitude of 1140 m above mean sea level (geographical coordinates 0° 18′ 49.18″ N latitude and 32° 36′ 43.86″ E longitude) (UBOS 2013). Kampala’s climate is tropical with precipitation throughout the year, mainly concentrated during two rainy seasons: the main one occurring between March and May and the second one in October and November. The driest month is July, which nevertheless receives an average of 62 mm precipitation (Climate-Data.org 2013).

**Fig. 1:**
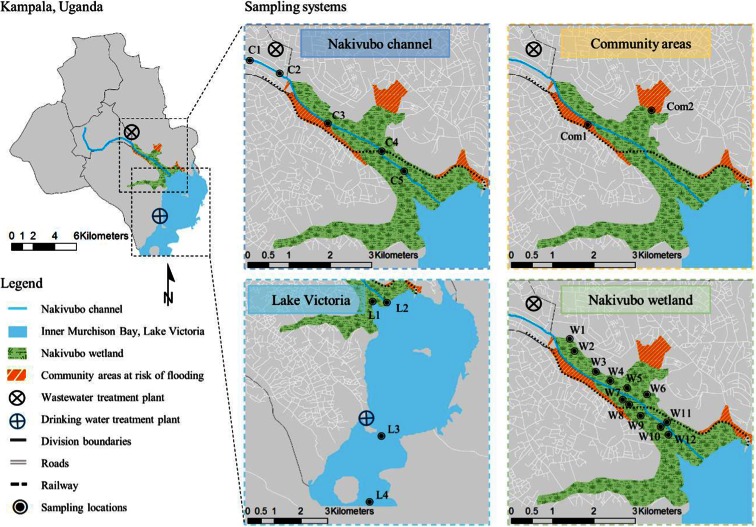
Map of Kampala showing the study area, including detailed maps of the four sampling systems with the specific sampling points (Nakivubo channel, Nakivubo wetland, community areas and Inner Murchison Bay in Lake Victoria)

The study area and the sampling scheme are shown in Fig. 1. For further details and a short video publication, the reader is referred elsewhere (Fuhrimann et al. 2014). In brief, the study area was divided into four sampling systems along the main wastewater chain of Kampala in the divisions Makindye and Nakawa; namely (i) the Nakivubo channel, (ii) the Nakivubo wetland, (iii) community areas bordering the wetland and (iv) the Inner Murchison Bay within Lake Victoria. The Nakivubo channel is 12.3 km long and transports wastewater from the communities, markets, industries and the secondary treated effluent from the BSTW until it drains into the Nakivubo wetland and, after another 4.5 km, reaches the Inner Murchison Bay of Lake Victoria. The Nakivubo wetland covers approximately 5.29 km^2^ and has a total catchment area of over 40 km^2^ (Emerton 1998). An old railway line divides the wetland into a northern and a southern part. North of the railway line is mainly drained farmland and the southern part is a floating wetland. Both areas are cultivated for yams and sugar cane. Informal communities that are at high risk of flooding are situated on both sides of the wetland (approximate population 12,000 people) (Kayima et al. 2008; Mbabazi et al. 2010). The Inner Murchison Bay is economically important for fish production and supplies Kampala with drinking water, which is pumped from about 4 km from the outlet of the Nakivubo channel.

### Sampling strategy

A cross-sectional survey was conducted between October and December 2013. As shown in Fig. 1, a total of 23 sentinel sites were selected, as follows:

- Nakivubo channel: five sampling points (C1–C5) spread over a distance of 4.5 km, starting above the inflow of the BSTW until Inner Murchison Bay. Water samples were taken at 16 time points, twice a week, whereas sediment samples were taken at two time points early and late during the study period.
- Nakivubo wetland: 12 sampling points (W1–W12) in four clusters where urban farming activities are pursued. Within the clusters, a stratified random sampling procedure was applied using a grid of 50 × 50 m (Webster and Lark 2013). Water, soil, and plant samples were taken at four time points, every second week.
- Community areas at risk of flooding: two sampling points (Co1 and Co2). Water and soil samples were collected at four time points, every second week.
- Shores of Lake Victoria: four sampling points (sampling at surface (*s*) and bottom (*b*)) within the Inner Murchison Bay (L1*s* and L2*s* and L1*b* and L2*b*), outlet of the Nakivubo channel; L3*s* and L3*b* in close proximity to drinking water treatment plant Ggaba II; and L4*s* and L4*b* reference point in the Inner Murchison Bay. Water samples were collected at eight time points, weekly, whereas sediment samples were taken at two time points at the beginning and towards the end of the study period.

### Sample collection

Water and sediment samples were collected in sterile wide mouth, screw-capped 1 L plastic bottles. Soil samples were collected in 2 L polyethylene bags. Plant (sugar cane and yams) samples were collected as whole plants. Samples were collected in the morning hours and transferred to a nearby laboratory in a cool box at a temperature of 4 °C.

### Physico-chemical analysis

While collecting the water samples, temperature, pH and electrical conductivity (EC) were measured in situ in the field using a Mettler-Toledo pH and EC meter (Mettler-Toledo International, Inc.; Greifensee, Switzerland). The following physico-chemical parameters were determined, adhering to standard methods (APHA, AWWA and WEF 2005): alkalinity (titrimetric method), total phosphate (persulphate method), orthophosphate (ascorbic method), ammonia-N (nesslerisation), nitrate-N (cadmium reduction spectrophotometric method), total solid suspended (TSS; photometric method), biochemical oxygen demand (BOD_5_; azide modification of the Winkler method; oxygen by the electrode method) and chemical oxygen demand (COD; closed reflux colorimetric method).

### Microbial analysis

All water samples were examined for TTC bacteria, *Escherichia coli*, *Salmonella* spp. and helminth eggs (*Ascaris lumbricoides*, hookworm and *Trichuris trichiura*), using standard protocols recommended by the World Health Organization (WHO) (Mara and Horan 2003; WHO 2004). For bacteria examination, a membrane filtration method was applied. Briefly, sample dilutions ranging from 10- to 100-fold were prepared and inoculated on membrane lauryl sulphate broth to count TTCs and *E. coli* and on xylose lysine deoxycholate (XLD) agar for *Salmonella* spp. Incubation temperatures and times were as follows: (i) TTCs 12–18 h at 44 °C, (ii) *E. coli* 18–22 h at 37 °C and (iii) *Salmonella* spp. 12–72 h at 37 °C (Ayres and Mara 1996). For the detection of helminth eggs, a modified Bailenger method was applied. The water or sediment samples (1 L each) were allowed to settle for a period of 12–15 h before the supernatant was drained off. Soil samples (250 g) were first diluted with 10 L of distilled water. Subsequently, the preparations were filtered for removing larger particles and then settled for 12–15 h. Water, sediment and soil samples were further analysed for helminth eggs with the McMaster method using acetoacetic buffer (pH 4.5) and zinc sulphate solution (specific density 1.18) (Ayres and Mara 1996; WHO 2004; Ensink 2006).

### Heavy metal analysis

Heavy metals were only measured at one time point during the assessment at all sentinel sites. In water, soil, sediment and eatable parts of plant samples, we analysed Cd, chromium (Cr), copper (Cu), Pb, iron (Fe) and zinc (Zn) by atomic absorption spectrophotometry (PerkinElmer2380, PerkinElmer Corporation; Norwalk, USA). For analysis of water samples, 100 mL of acid-preserved samples were processed using nitric acid digestion before carrying out spectrometric measurements. At least three calibrations with different dilutions of the relevant standard solutions were done beforehand (APHA, AWWA and WEF 2005). Soil, sediment and plant samples were dried for 24 h at 105 °C, ground to fine powder and digested with mineral acids and the resultant solutions analysed by atomic absorption spectrophotometry as detailed in Mbabazi et al. (2010).

### Statistical analysis

The software R, version 3.0.2, was used for statistical analyses (The R Foundation for Statistical Computing; Vienna, Austria). A log-normal probability density function was applied for characterisation of pathogen concentrations in water. Geometric mean concentrations, including 95 % confidence intervals (CIs) were calculated using a Student’s *t* test. Data on the contamination of the Nakivubo wetland were compared with WHO guidelines for the safe use of wastewater in agriculture (WHO 2006) and with standards for the discharge of effluents into water or on land developed by the Ugandan National Environmental Management Authority (NEMA) (NEMA 1999).

## Results and discussion

### Physico-chemical parameters in water

**Fig. 2:**
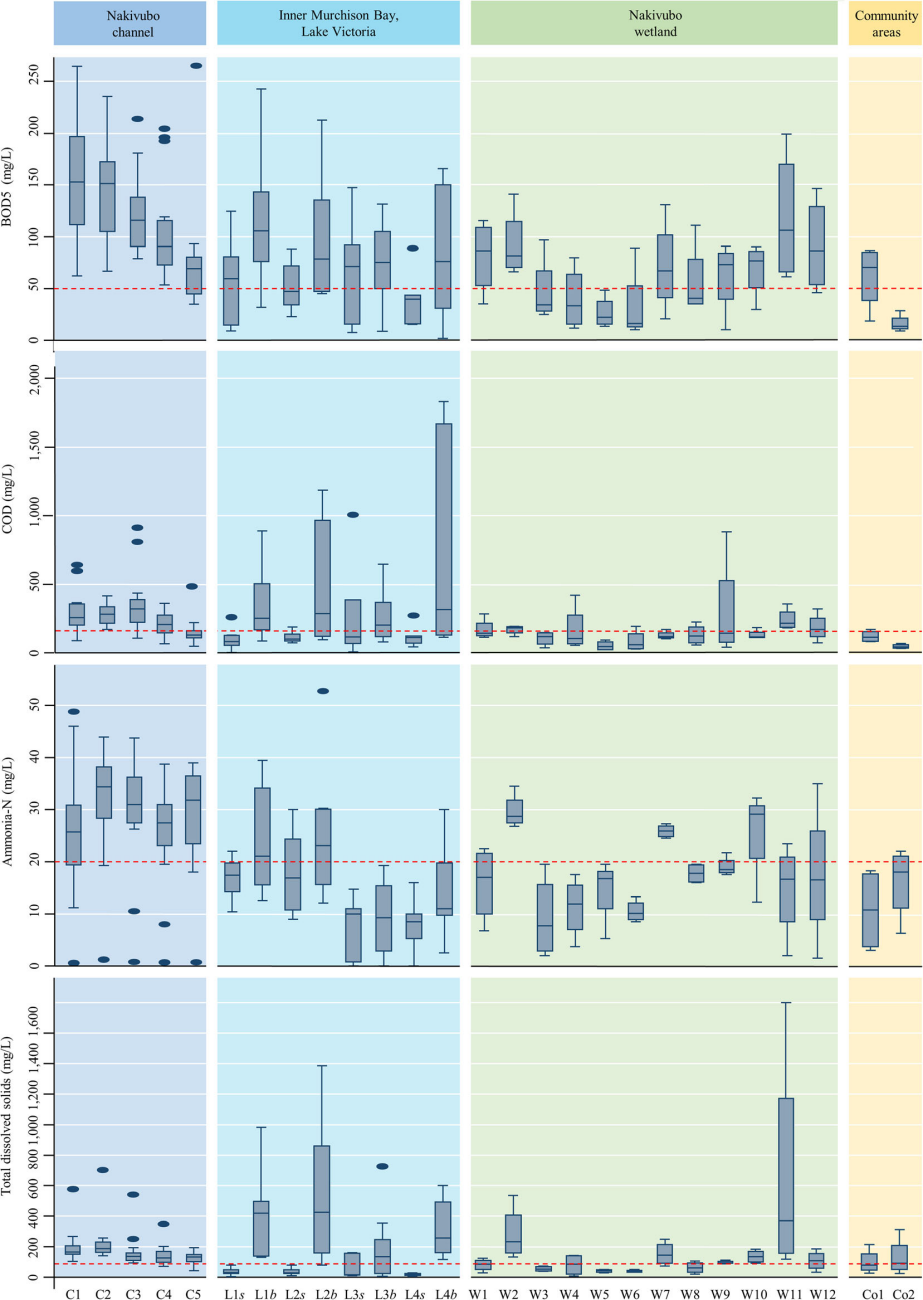
Box-and-whisker plot of the concentration of BOD_5_, COD, ammonia-N and total solid suspended in the four sampling systems along the Nakivubo channel, in Kampala, Uganda (sampling period: 15 October to 5 December 2013). *Red line*: maximum acceptable concentrations of TTCs (NEMA 1999) and *E. coli* (WHO 2006). L*s* surface and L*b* bottom samples taken within the Inner Murchison Bay of Lake Victoria

Table 1 summarises the physico-chemical parameters of water samples, including temperature, pH, EC, total alkalinity, TSS, BOD_5_, COD, total phosphate, orthophosphate, ammonia-N and nitrate-N. As expected, the highest values of the investigated physico-chemical parameters were found in the Nakivubo channel, while the lowest values were obtained from the samples taken in the Inner Murchison Bay. Figure 2 shows BOD_5_, COD, TSS and ammonia-N for each sampling point, presented as box plots. For BOD_5_, there was a decrease along the Nakivubo channel from a median value of 156.7 mg/L (C1—furthest from Lake Victoria) to about 75.9 mg/L (C5—nearest to the lake). The values for the other parameters only decreased minimally.

**Table 1:** Physico-chemical parameters of water samples in the four sampling systems along the Nakivubo channel in Kampala (sampling period: 15 October to 5 December 2013)

Physico-chemical parameter	Min	Max	Mean	Lower 95 % CI	Upper 95 % CI	NEMA standards
Temperature (°C)	18.1	34.3	26.4	26.1	26.8	20.0–35.0
pH	5.9	9.3^a^	7.2	7.1	7.3	6.0–8.0
EC (μS/cm)	104.7	1320.0	574.6	538.1	611.2	1500.0
Total alkalinity (mg/L)	28.0	556.0	240.5	225.1	255.8	800.0
TSS (mg/L)	6.0	5100	198.7^a^	140.8^a^	256.7^a^	100.0
BOD_5_ (mg/L)	2.0	425.7	91.4^a^	82.7^a^	100.0^a^	50.0
COD (mg/L)	5.0	3231^a^	257.4^a^	211.3^a^	303.5^a^	100.0
Total phosphate (mg/L)	0.01	84.1^a^	11.5^a^	9.7	13.3^a^	10.0
Orthophosphate (mg/L)	0.0	26.2^a^	5.2^a^	4.5	5.9	5.0
Ammonia-N (mg/L)	0.0	52.8^a^	21.2^a^	19.6^a^	22.8^a^	10.0
Nitrate-N (mg/L)	0.0	2.5	0.2	0.15	0.25	10.0

The minimum and maximum concentration, geometric mean and 95 % confidence intervals (CIs) for Student’s *t* test are indicated

^a^Concentrations exceeding maximum acceptable concentrations (NEMA 1999)

The physico-chemical parameters of the water samples showed large spatial heterogeneity. The lower levels in the Inner Murchison Bay can be explained by dilution after discharge into Lake Victoria (Kansiime and Nalubega 1999). The comparison of the geometric means with standard values set by NEMA revealed biogenic pollution; the mean values for BOD_5_, COD, TSS, ammonia-N and total phosphate all exceeded the national standards for the discharge of effluents into the environment. When compared with data published in 2008 for the Nakivubo channel (Kayima et al. 2008), our results suggest an increase of up to 200–300 %. In view of these findings, control measures, such as the channelisation of the Nakivubo channel that was done in 2008, seem to have failed to reduce environmental pollution (Beller et al. 2004).

### Bacterial parameters in water

**Fig. 3:**
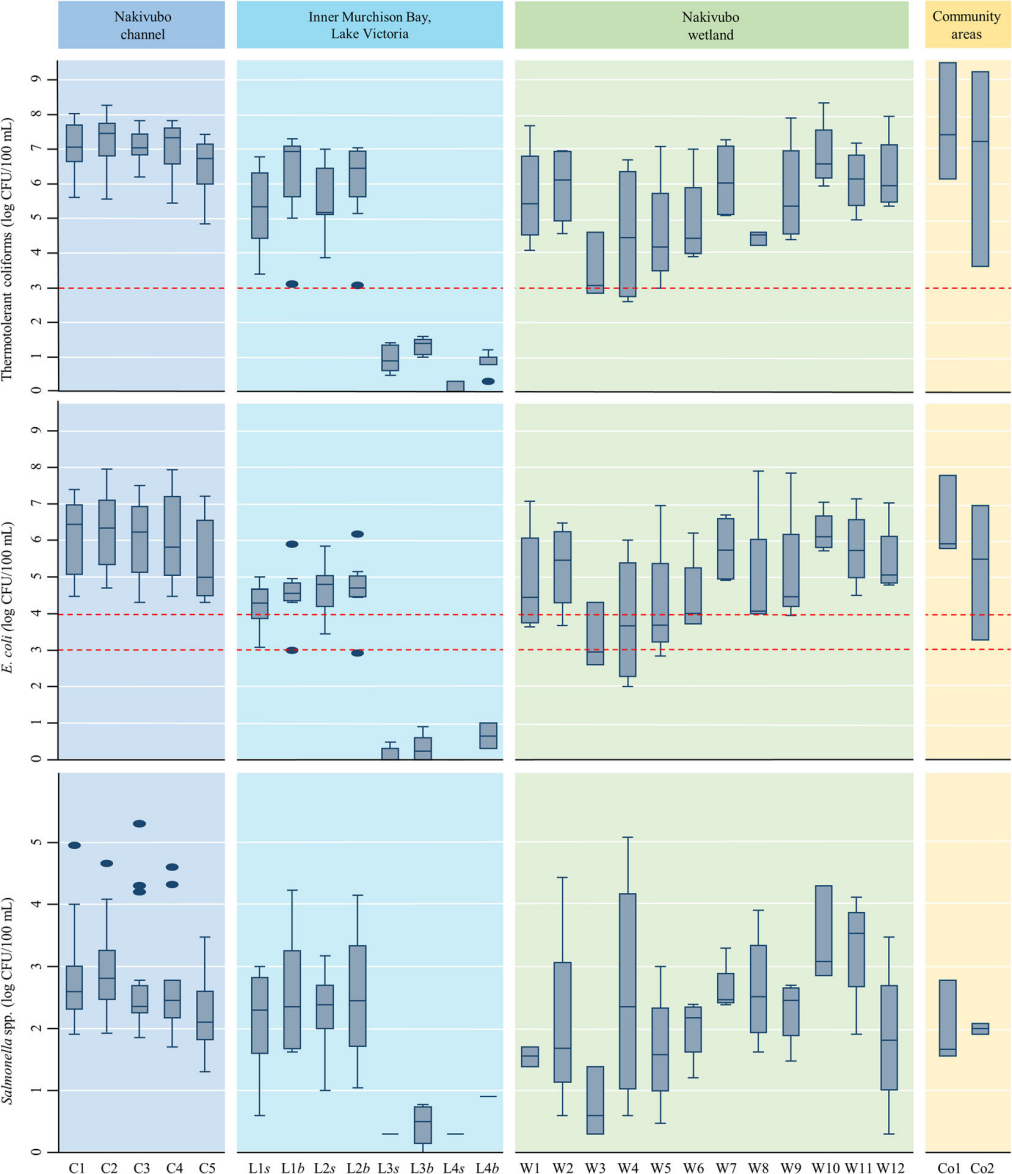
Box-and-whisker plot of the concentration of thermotolerant coliforms (TTCs), *E. coli* and *Salmonella* spp. in the four sampling systems along the Nakivubo channel, in Kampala (sampling period: 15 October to 5 December 2013). *Red line*: maximum acceptable concentrations of TTCs (NEMA 1999) and *E. coli* (WHO 2006). L*s* surface and L*b* bottom samples taken within the Inner Murchison Bay of Lake Victoria

The results of TTCs, *E. coli* and *Salmonella* spp. in water samples obtained from the four sampling systems are displayed in Table 2. TTC and *E. coli* concentrations were highest in the samples taken within the community areas and ranged from 4.0 × 10^2^ to 2.2 × 10^8^ and from 1.0 × 10^2^ to 7.9 × 10^7^ colony-forming units (CFU)/100 mL, respectively. *Salmonella* spp. was found in all sampling points with a mean concentration of 3.8 × 10^2^ CFU/100 mL in the Nakivubo channel and 1.3 CFU/100 mL in Lake Victoria. Figure 3 shows that there was a decrease in bacteria concentrations along the channel with increasing distance from Kampala city, both for TTCs and *E. coli*. However, this trend was interrupted by an increase in bacteria concentrations between the sampling point just before and after the inlet of BSTW’s effluent, which indicates additional contamination by the treatment plant. The natural treatment function of the wetland was only obvious for *E. coli*; from the beginning of the floating wetland (C4) to the discharge of the Nakivubo channel in Lake Victoria (L1*s*/*b* and L2*s*/*b*), the mean concentration decreased by 1.56 log CFU.

**Table 2:** Thermotolerant coliforms, *E. coli* and *Salmonella* spp. concentrations in the four sampling systems along the Nakivubo channel in Kampala (sampling period: 15 October to 5 December 2013)

Sampling system	Counts in CFU/100 mL				
Bacteria in water	Min	Max	Mean	Lower 95 % CI	Upper 95 % CI
Nakivubo channel (*n* = 112)					
Thermotolerant coliforms	1.2 × 10^3^	1.8 × 10^8^	4.3 × 10^6a^	2.7 × 10^6a^	6.9 × 10^6a^
*E. coli*	8.4 × 10^2^	9.0 × 10^7^	3.8 × 10^5a^	2.3 × 10^5a^	6.4 × 10^5a^
*Salmonella* spp.	0.0	2.0 × 10^5^	3.8 × 10^2^	2.5 × 10^2^	5.7 × 10^2^
Nakivubo wetland (*n* = 48)					
Thermotolerant coliforms	4.0 × 10^2^	2.2 × 10^8^	2.9 × 10^5a^	1.0 × 10^5a^	8.0 × 10^5a^
*E. coli*	1.0 × 10^2^	7.9 × 10^7^	9.9 × 10^4a^	3.6 × 10^4a^	2.7 × 10^5a^
*Salmonella* spp.	0.0	1.2 × 10^5^	1.4 × 10^2^	63.0	3.2 × 10^2^
Community areas (*n* = 8)					
Thermotolerant coliforms	4.2 × 10^3^	3.1 × 10^9^	1.5 × 10^7a^	8.4 × 10^4^	2.9 × 10^9a^
*E. coli*	1.9 × 10^3^	6.0 × 10^7^	7.3 × 10^5a^	1.8 × 10^4^	2.9 × 10^7a^
*Salmonella* spp.	36.0	6.0 × 10^2^	99.0	35.0	2.8 × 10^2^
Lake Victoria (*n* = 32)					
Thermotolerant coliforms	0.0	40.0	3.7	2.3	6.0
*E. coli*	0.0	11.0	1.3	1.1	1.7
*Salmonella* spp.	0.0	8.0	1.3	1.0	1.6

The minimum and maximum concentration, geometric mean and 95 % confidence intervals (CIs) for Student’s *t* test are indicated

^a^Concentrations exceeding maximum acceptable concentrations for faecal coliforms (NEMA 1999) and *E. coli* (WHO 2006)

Our findings show much higher microbial contamination than previously reported for Kampala (Kayima et al. 2008) and might underline a decrease in the natural treatment function of the Nakivubo wetland (Kansiime and Nalubega 1999). Flooding events of the Nakivubo channel and wetland may also contribute to the pollution of protected springs, as indicated by Nsubuga et al. (2004). Hence, our results are in line with results recently reported for open storm drainage channels and the Bwaise III slums areas in Kampala (Katukiza et al. 2014). They even showed for open drainage channels mean values for CFU *E. coli* and *Salmonella* spp. per 100 mL of up to 7.9 × 10^6^ and 2.0 × 10^5^, respectively. Mean concentrations for TTCs of up to 1.5 × 10^7^ CFU/100 mL in the two sampling points close to the community areas exceeded the national thresholds for wastewater discharge of 5.0 × 10^3^ CFU/100 mL by more than 4 log. When comparing the mean TTC concentration at railway bridge (C4) with results published for the wet season in 2008 (Kayima et al. 2008), we found 3.1 log higher concentrations. Only TTC concentrations in water from Lake Victoria were below the current effluent discharge standards. Results of our study also found mean concentrations of up to 3.8 × 10^5^ and 7.3 × 10^5^ CFU *E. coli*/100 mL in the water of the Nakivubo channel. In the Nakivubo wetland, WHO thresholds for unrestricted irrigation were exceeded, as we found that values were above the recommended verification limits of 10^3^–10^4^ CFU *E. coli*/100 mL (Table 2, Fig. 3). Such high concentrations of these bacteria may result in adverse health impacts among farmers and community members, who are directly or indirectly exposed to these waters. For the safety of these population groups, additional control measures are required (WHO 2006). The fact that we measured low concentration of TTCs, *E. coli* and *Salmonella* spp. at L3 and L4 in the Murchison Bay of Lake Victoria should be considered for future monitoring of drinking water quality. As these bacteria mainly serve as indicator organisms for faecal pollution, the source for drinking water in Kampala is likely to be polluted by pathogenic bacteria, viruses and protozoa organism, which may survive the treatment processes (Ayres and Mara 1996; Howard et al. 2006; WHO 2011).

### Contamination of water, sediment and soil with helminth eggs

Table 3 shows that 15.5 % of all water samples were positive for helminth eggs; 13.5 % for hookworm and 2.0 % for *A. lumbricoides*, whilst no *T. trichiura* eggs were detected. In eight sediment samples along the Nakivubo channel, hookworm and *A. lumbricoides* eggs were found (12.5 %) in the 28 soil samples collected in the Nakivubo wetland. Hookworm eggs were recovered in 14.3 % of the samples, while neither *A. lumbricoides* nor *T. trichiura* eggs were found.

**Table 3:** Helminth egg counts (hookworm and *Ascaris lumbricoides*) and prevalence in the four sampling systems in Kampala obtained from water samples (sampling period: 15 October to 5 December 2013)

Sampling system	Egg counts (/L)					No. positive	Prevalence rates		
Helminth egg in water	Min	Max	Mean	Lower 95 % CI	Upper 95 % CI		% positive	Lower 95 % CI	Upper 95 % CI
Nakivubo channel (*n* = 112)									
Hookworm	0	160	2.0	1.5	2.6	23	20.5	13.0	28.0
*Ascaris lumbricoides*	0	10	1.1	1.0	1.1	3	2.7	–	–
Nakivubo wetland (*n* = 48)									
Hookworm	0	933	1.3	0.9	1.8	3	6.3	–	–
*Ascaris lumbricoides*	0	0	–	–	–	0	0	–	–
Community areas (*n* = 8)									
Hookworm	0	0	–	–	–	0	0	–	–
*Ascaris lumbricoides*	0	40	1.6	0.5	4.7	1	12.5	–	–
Lake Victoria (*n* = 32)									
Hookworm	0	40	1.3	0.8	2.2	1	3.1	–	–
*Ascaris lumbricoides*	0	0	–	–	–	0	0	–	–
Total (*n* = 200)									
Hookworm						27	13.5	8.8	18.2
*Ascaris lumbricoides*						4	2.0	0.1	3.9
Total helminth eggs						31	15.5	10.5	20.5

For egg counts, the minimum and maximum, geometric mean and 95 % confidence intervals (CIs) for Student’s *t* test are indicated. For the prevalence, CIs are indicated

Overall, the mean concentration of helminth eggs in water samples was between 1.3 and 2 eggs per L and therefore exceeds the WHO guidelines for the safe use of wastewater (<1 egg per L). It follows that additional control measures are required to protect people who are in frequent contact with this water (WHO 2006). The highest mean helminth egg counts were found in water samples in the Nakivubo channel. *A. lumbricoides* eggs were detected in water samples obtained from the Nakivubo channel, but not from the Nakivubo wetland, suggesting a natural treatment function of the wetland (Jimenez-Cisneros 2006). It is conceivable that this treatment function also applies to hookworm eggs (Stott et al. 2003). However, as hookworm eggs were found in the wetland, we speculate that there is continuous contamination of the wetland with hookworm eggs. As our study was conducted during the second, short rain season of 2013 (between October and December), different prevalence rates for the first and longer rainy season (March–May) might be expected (Motazedian et al. 2006).

### Contamination of water, sediments, soils and plants with heavy metals

As shown in Table 4, highest heavy metal contaminations were found in water sampled in the Nakivubo channel and within the community areas. Data presented in Table 5 summarise the heavy metal concentration in sediment of the Nakivubo channel and in soil and plant samples taken from the Nakivubo wetland. In the Nakivubo channel, the geometric means for Fe, Cu and Cd in water samples were 21.5, 3.3 and 0.14 mg/L, respectively, and exceeded the effluent discharge standards by NEMA. Regarding Cr, the upper 95 % CI of the concentration in water from the wetland area was above the maximum acceptable concentrations (MACs).

**Table 4:** Concentration of heavy metals (copper (Cu), zinc (Zn), iron (Fe), cadmium (Cd), lead (Pb) and chromium (Cr)) in water in the four sampling systems along the Nakivubo channel in Kampala (sampling period: 18 and 19 November 2013).

Sampling system	Concentration in mg/L					
Heavy metals	Min	Max	Mean	Lower 95 % CI	Upper 95 % CI	Guideline values
Nakivubo channel (*n* = 5)						
Cu	0.90	6.30	3.30^a^	1.60^a^	5.00^a^	1.00
Zn	0.20	3.00	1.40	0.70	2.00	5.00
Fe	8.10	38.10^a^	21.50^a^	13.90^a^	29.00^a^	10.00
Cd	0.05	0.31^a^	0.14^a^	0.07^a^	0.22^a^	0.10
Pb	0.09	3.00^a^	1.60^a^	0.94^a^	2.26^a^	0.10
Cr	0.01	0.01	0.06	0.03	0.08	1.00
Nakivubo wetland (*n* = 12)						
Cu	0.90	4.00^a^	2.30^a^	1.70^a^	3.00^a^	1.00
Zn	0.01	1.10	0.30	0.10	0.60	5.00
Fe	10.90^a^	33.50^a^	18.60^a^	14.10^a^	23.10^a^	10.00
Cd	0.01	0.31^a^	0.13^a^	0.07	0.19^a^	0.10
Pb	0.10	2.60^a^	1.02^a^	0.56^a^	1.49^a^	0.10
Cr	0.003	0.21	0.06	0.01	0.10	1.00
Community areas (*n* = 2)						
Cu	1.70^a^	4.20^a^	3.00^a^	–	–	1.00
Zn	0.17	0.20	0.19	–	–	5.00
Fe	18.20^a^	27.60^a^	22.90^a^	–	–	10.00
Cd	0.14^a^	0.26^a^	0.20^a^	–	–	0.10
Pb	1.30^a^	3.80^a^	2.50^a^	–	–	0.10
Cr	0.01	0.02	0.02	–	–	1.00
Lake Victoria (*n* = 4)						
Cu	1.00	2.10^a^	1.40^a^	0.60^a^	2.20^a^	1.00
Zn	0.20	0.50	0.30	0.10	0.50	5.00
Fe	15.00^a^	21.10^a^	17.70^a^	12.70^a^	22.70^a^	10.00
Cd	0.09	0.11^a^	0.10	0.09^a^	0.11^a^	0.10
Pb	0.91^a^	1.64^a^	1.25^a^	0.76^a^	1.73^a^	0.10
Cr	0.01	0.02	0.014	0.004	0.023	1.00

Geometric means and 95 % confidence intervals (CIs) are indicated

^a^Concentrations exceeding maximum acceptable concentrations (MACs) as per Ugandan guidelines (NEMA 1999)

**Table 5:** Concentration of heavy metals (copper (Cu), zinc (Zn), iron (Fe), cadmium (Cd), lead (Pb), and chromium (Cr)) in sediment, soil and plants (yam and sugar cane) in along the Nakivubo channel, in Kampala (sampling period: 18 and 19 November 2013)

Sample type	Concentration in mg/L					
Heavy metals	Min	Max	Mean	Lower 95 % CI	Upper 95 % CI	Guideline values
Sediment from the Nakivubo channel and Lake Victoria (*n* = 8)						a
Cu	12.80	78.30	35.80	16.90	54.80	100.00
Zn	37.00	351.30	134.90	35.40	234.40	300.00
Fe	15,000	28,000	25,000	20,000	30,000	50,000
Cd	0.50	5.30^a^	2.10	0.90	3.30^a^	3.00
Pb	2.50	90.00	25.60	2.20	49.00	100.00
Cr	29.00	103.00^a^	49.80	30.70	68.90	100.00
Soil form the Nakivubo wetland and community areas (*n* = 28)						a
Cu	18.30	98.30	53.10	44.30	61.80	100.00
Zn	32.00	742.50^a^	293.00	218.00	368.00^a^	300.00
Fe	15,000	80,000^a^	47,000	40,000	54,000^a^	50,000
Cd	0.30	3.50	1.80	1.50	2.10	3.00
Pb	20.00	427.50^a^	132.70^a^	98.40^a^	167.00^a^	100.00
Cr	24.50	105.30^a^	49.40	41.20	57.50	100.00
Yam (*n* = 15)						b
Cu	0.00	11.90	2.60	0.70	4.50	73.00
Zn	0.00	387.50^a^	62.80	5.10	120.50^a^	100.00
Fe	0.00	87.50	42.10	23.70	60.50	425.00
Cd	0.00	0.50^a^	0.20^a^	0.10	0.30^a^	0.10
Pb	0.00	8.80^a^	4.00^a^	2.20^a^	6.00^a^	0.30
Cr	0.00	13.90^a^	4.40^a^	30.70^a^	7.10^a^	2.30
Sugarcane (*n* = 13)						b
Cu	0.00	9.00	2.40	0.80	4.00	73.00
Zn	0.00	553.80	67.10	–	–	100.00
Fe	26.30	92.50	59.00	47.20	70.70	425.00
Cd	0.00	0.50^a^	0.20^a^	0.10	0.30^a^	0.10
Pb	0.00	17.50^a^	4.30^a^	1.20^a^	7.50^a^	0.30
Cr	1.00	14.30^a^	8.40^a^	5.40^a^	11.50^a^	2.30

Geometric means and 95 % confidence intervals (CIs) are indicated

^a^Concentrations exceeding maximum acceptable concentrations (MACs) as per guideline values (a, FAO (2004) and b, FAO/WHO (2001))

Our findings for Cu, Cd, Pb and Zn measured in the Nakivubo wetland and Lake Victoria are in line with previous studies conducted in the same area (Barifaijo et al. 2009; Mbabazi et al. 2010). However, the present study shows considerably lower heavy metal concentrations at the beginning of the Nakivubo channel. This observation might be explained by temporal variation (Mbabazi et al. 2010). It should also be noted that the sample size was small (one sample per sampling point), and hence, care is indicated while interpreting our findings.

In soil and sediment samples, only the mean concentration of Pb exceeded the MAC. Taking the lower 95 % CI into account, values for Fe, Cd and Zn exceeded the standards. Nevertheless, the levels are well below the stated intervention levels by FAO (2004). In the examined plant samples (yams and sugar cane), mean concentrations of Cd, Cr and Pb (yams 4.4, 4.0 and 0.2 mg/L; sugar cane 8.4, 4.3 and 0.2 mg/L, respectively) exceeded thresholds put forth by NEMA (1999). The upper 95 % CI of Zn levels in yams showed an elevated concentration of 120.5 mg/L (Table 5) (FAO/WHO 2001).

Soil and plant samples showed similar results to those obtained in the Nakivubo wetland by Sekabira et al. (2011). In comparison with a recent study conducted in Accra, Ghana, which focused on wastewater-irrigated vegetables (Ackah et al. 2013), the heavy metal levels in our soil and sediment samples were considerably higher. Particularly, the high values of Pb and Cd in the plant samples are of public health concern as they may accumulate in body tissue and cause adverse chronic health effects (Mwegoha and Kihampa 2010; Abaidoo et al. 2010). The tracing of heavy metals throughout the food chain, including other food crops, vegetables and also fish in Lake Victoria should be considered, to assess the related public health burden (Birungi et al. 2007; Ackah et al. 2013). Hence, to obtain a more complete picture of chemical pollution in Kampala’s wastewater, studies—including other hazardous chemicals such as pesticides, pharmaceuticals, endocrine disruptors and illicit drugs— are needed (Belgiorno et al. 2007; Fatta-Kassinos et al. 2011).

### Potential and effective health risks for exposed groups along the wastewater chain

This environmental sampling was part of a larger study, comprising of a cross-sectional parasitological survey in selected population groups, a quantitative microbial risk assessment to determine the health risks related to microbial contamination, and the development and validation of a sanitation safety planning (SSP) manual (Fuhrimann et al. 2014). The results of our environmental monitoring (particularly contamination of water, sediment and soil with helminth eggs) are particularly interesting when juxtaposed with results obtained from a cross-sectional parasitological survey conducted in the Nakivubo area, which focused on five different population groups. With regard to intestinal parasitic infections (helminths and intestinal protozoa), farmers were found at the highest risk (overall prevalence of infection 75.9 %), followed by exposed community members (53.2 %), non-exposed community members (44.7 %), wastewater treatment plant workers (41.9 %) and faecal sludge collectors (35.8 %) (Fuhrimann et al. 2014).

## Conclusions

This study revealed considerable microbial and chemical contamination of the Nakivubo wetland and Lake Victoria ecosystem due to domestic and industrial wastewater flows through Kampala City. A decrease in bacteria concentrations along the wetland was observed only for *E. coli* and BOD_5_. Our sampling took place over an 8-week period between October and December 2013, corresponding to the rainy season. Therefore, our findings represent a snapshot of microbial and chemical contamination in the rainy season along the wastewater chain.

Our findings make an important contribution to the understanding of the nexus of wastewater pollution and its direct implications for public health in the context of a major East African city in the Great Lake region. We propose that a system of sentinel monitoring is established that will inform evidence-based decision-making and responses that are readily tailored to this social-ecological system. Our study suggests that farmers, consumers of plants grown in the Nakivubo wetland and communities living in close proximity to areas prone to flooding are exposed not only to high loads of pathogenic bacteria but also to helminth eggs (mainly hookworm) and heavy metals. Efforts should therefore be made by local authorities to minimise risks for these population groups by applying control measures (both technical and non-technical).
